# Comparison of Genetic Profiling between Primary Tumor and Circulating Tumor Cells Captured by Microfluidics in Epithelial Ovarian Cancer: Tumor Heterogeneity or Allele Dropout?

**DOI:** 10.3390/diagnostics11061102

**Published:** 2021-06-16

**Authors:** Ting-Yu Chang, Sheng-Wen Chen, Wen-Hsiang Lin, Chung-Er Huang, Mark I. Evans, I-Fang Chung, Janne-Wha Wu, Gwo-Chin Ma, Ming Chen

**Affiliations:** 1 Department of Genomic Medicine, Changhua Christian Hospital, Changhua 50046, Taiwan; taiwanbird@gmail.com; 2 Department of Research, Changhua Christian Hospital, Changhua 50006, Taiwan; 3 Department of Bioscience Technology, Chung Yuan Christian University, Taoyuan 32023, Taiwan; 4 Department of Electrical Engineering, National Chung Cheng University, Chiayi 62102, Taiwan; ivan.chen@cytoaurora.com; 5 Cytoaurora Biotechnologies Inc., Hsinchu Science Park, Hsinchu 30261, Taiwan; ce.huang@cytoaurora.com; 6 Welgene Biotechnology Company, Nangang Business Park, Taipei 11503, Taiwan; 397620cch@gmail.com; 7 Comprehensive Genetics, New York, NY 10065, USA; evans@compregen.com; 8 Department of Obstetrics and Gynecology, Icahn School of Medicine at Mt. Sinai, New York, NY 10029, USA; 9 Institute of Biomedical Informatics, National Yang Ming Chiao Tung University, Taipei 11221, Taiwan; ifchung@ym.edu.tw; 10 Department of Communications Engineering, National Chung Cheng University, Chiayi 62102, Taiwan; 11 Department of Biomedical Engineering, Chung Yuan Christian University, Taoyuan 32023, Taiwan; 12 Department of Obstetrics and Gynecology, College of Medicine and Hospital, National Taiwan University, Taipei 100225, Taiwan; 13 Department of Biomedical Science, Dayeh University, Changhua 515006, Taiwan; 14 Department of Medical Sciences, National Tsing Hua University, Hsinchu 300044, Taiwan

**Keywords:** EOC, CTC, liquid biopsy, whole exome sequencing, tumor heterogeneity, allele dropout

## Abstract

Epithelial ovarian cancer (EOC) is a leading cause of cancer mortality among women but unfortunately is usually not diagnosed until advanced stage. Early detection of EOC is of paramount importance to improve outcomes. Liquid biopsy of circulating tumor cells (CTCs) is emerging as one of the promising biomarkers for early detection of solid tumors. However, discrepancies in terms of oncogenomics (i.e., different genetic defects detected) between the germline, primary tumor, and liquid biopsy are a serious concern and may adversely affect downstream cancer management. Here, we illustrate the potential and pitfalls of CTCs by presenting two patients of Stage I EOC. We successfully isolated and recovered CTCs by a silicon-based nanostructured microfluidics system, the automated Cell Reveal^TM^. We examined the genomics of CTCs as well as the primary tumor and germline control (peripheral blood mononuclear cells) by whole exome sequencing. Different signatures were then investigated by comparisons of identified mutation loci distinguishing those that may only arise in the primary tumor or CTCs. A novel model is proposed to test if the highly variable allele frequencies, between primary tumor and CTCs results, are due to allele dropout in plural CTCs or tumor heterogeneity. This proof-of-principle study provides a strategy to elucidate the possible cause of genomic discrepancy between the germline, primary tumor, and CTCs, which is helpful for further large-scale use of such technology to be integrated into clinical management protocols.

## 1. Introduction

Ovarian cancer (OC) is one of the most lethal gynecologic malignancies in the developed world. They are divided into three groups: epithelial, germ cell, and specialized stromal cell tumors; the epithelial ovarian cancer (EOC) is the most common [1]. The OC is usually not diagnosed until advanced stage, in which dissemination has already occurred. Conventional diagnosis of OC relies on surgical recovery of tissue which is only justified when clinical or poor to mediocre screening tests suggest suspicion for malignancy, commonly achieved by biomarkers (e.g., elevated serum CA-125) and image modalities (e.g., transvaginal ultrasound) [2]. This strategy shows disadvantages of poor screening performance, late diagnosis, and limited treatment statistics, and thus alternative approaches are demanded. Getting genomic information of solid tumor through minimally invasive peripheral blood testing to facilitate early detection has the potential when standardized to guide subsequent treatments [3,4]. Currently, advanced technologies such as next generation sequencing (NGS) has made “liquid biopsy” possible.

Liquid biopsy is the sampling and analysis of nonsolid biological specimens, such as blood and body fluids. It allows rapid biomarker assessment in cancer patients, facilitating early diagnosis, risk prediction, and cancer classification. There are several modalities of liquid biopsies, such as circulating cell-free tumor DNA (ctDNA), circulating tumor cells (CTCs), circulating miRNA, circulating RNA, plasma/serum metabolites, and exosomes [5,6,7]. Among these modalities, genetic analyses of ctDNA and CTCs from blood samples have been tentatively tested in OC [8,9]. The ctDNA and CTCs are released into the blood by multiple mechanisms, including tumor cell necrosis, apoptosis, and lysis [5].

The mutation spectrums have been investigated in a number of cancers, including OC. Genetic alternations in *TP53*, *NF1*, *BRCA1*, *BRCA2*, *RB1*, *CDK12*, and *CCNE1* as well as epigenetic changes in multiple loci are frequent in OC [8,10]. The ctDNA is currently the mainstream modality compared to CTCs in liquid biopsy because CTCs lack the reliability and reproducibility of the ctDNA. However, with better analysis methods, CTCs might provide more information than the fragmented ctDNA [11]. Unfortunately, very few feasible platforms are currently available to isolate and recover CTCs [12]. Analogous to prenatal diagnosis for which fetal cells have the potential for enhanced capabilities as compared to cell free fetal DNA, CTCs might provide tissue-specific information which could be critical to ideal therapeutic options; for example, the detection of PDL1 in non-small cell lung cancer to serve as a biomarker for immunotherapy [11,13]. Targeted earlier diagnoses may identify some tumor-specific and tissue-specific proteomic antigens and can advance identification of the origin of the primary tumor [11,14,15,16,17].

Obtaining a specimen is only the first step towards developing a successful screening or diagnostic test. The highly heterogeneous nature of tumor genomics complicates interpreting genetic data. The heterogeneity may arise in the primary tumor itself, may exist between the primary tumor and the shed CTCs, and may even exist among the CTCs captured since they may originate from different parts of primary tumors [18]. Inherent errors arising from the experimental steps also hinder the utility of such data, therefore, whether a few CTCs can recapitulate the whole primary tumor remain uncertain. Both ctDNA and CTCs can detect tumor heterogeneity (either in spatial or temporal dimension) and the mechanisms/processes involved in metastasis, including the possible role of CTCs, had been reviewed and discussed [16,19,20]. However, whether CTCs is a suitable biomarker in liquid biopsy remains controversial since some researchers reported a better detection rate of mutations in the actionable genes such as *KRAS* and *EGFR* by ctDNA versus CTCs in colorectal cancer and lung cancer [21,22].

We have published an automated system, the Cell Reveal^TM^, involving multi-antibody-mediated approach for rare cells capture and retrieval [23,24,25]. The feasibility of differentiating the benign ovarian tumors and EOC by the Cell Reveal^TM^ system had been also shown in our recently published study, in which SKOV3 cell line was used for in vitro spike test and a test with 100% specificity to differentiate eight EOC patients from five patients with benign ovarian tumors was reached [25]. Here, we used the Cell Reveal^TM^ system for EOC retrieval, in which the CTCs can be captured before debulking surgery. Recovered cells underwent genomic analyses using whole genome amplification (WGA) followed by whole exome sequencing. Mutation spectrums of two patients with Stage I EOC (one epithelial and one endometrioid by tissue pathology) were reported, summarized, and analyzed by inferring the involved pathways in tumorigenesis. Since the genetic analysis of CTCs was performed with plural cells, rather than individual cells, a novel model was also proposed to test whether the variations of allele frequencies in mutated loci are due to allele dropout (ADO) in plural CTCs or tumor heterogeneity, one of the major sources of introduced artificial errors.

## 2. Materials and Methods

### 2.1. Patients and Clinical Information

In 2019, two patients with Stage I EOC underwent liquid biopsy to recover CTCs using a silicon-based nanostructured microfluidics system, the automated Cell Reveal^TM^ [23,24,25]. Patient 1 had serous adenocarcinoma with FIGO stage T_1C3_N_0_M_0_. Liquid biopsy was carried out before debulking surgery. At surgery, tumor cells were found in the left ovary and in peritoneal washings, but no tumor cells were noted in the resected uterus, right ovary, both fallopian tubes, omentum, the cul-de-sac biopsy site, and regional lymph nodes. Patient 2 had endometrioid adenocarcinoma of ovary with FIGO stage T_1A_N_0_M_0_. Liquid biopsy was also performed before debulking surgery. T cells were detected in the right ovary. Tumor cells were negative in the resected uterus, left side uterus, both fallopian tubes, omentum, and regional lymph nodes.

This study was approved by the Ethics Committee of the Changhua Christian Hospital, Changhua, Taiwan (project ID: CCH-IRB-190710 and CCH-IRB-190117). All participants gave written informed consent before the study began.

### 2.2. Tumor Tissue and Germline Control Correction

Tumor tissue was collected from the surgical excision by the dimension of 5 mm on each axis roughly. Pathological examination confirmed the majority of specimen to be malignant cells. Germline control DNA was obtained from peripheral blood mononuclear cells (PBMCs) of patients. Genomic DNA was purified using Qiagen DNeasy Blood and Tissue kit (Qiagen, CA, USA), according to the manufacturer’s instruction.

### 2.3. CTCs Capture and Recovery

**Figure 1:**
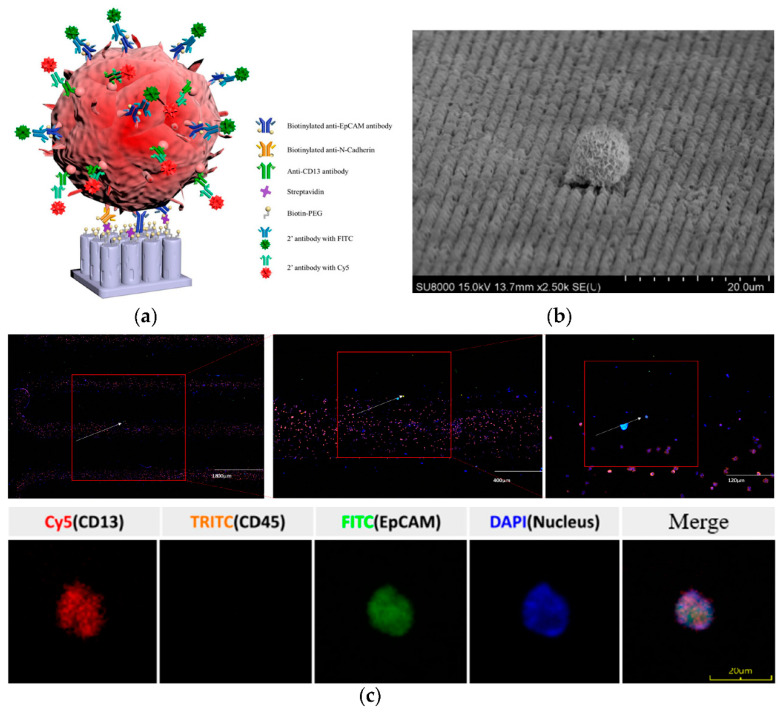
CTCs were captured by the automated Cell Reveal^TM^ system with a nano structure V-BioChip. (**a**) The principle of antibody mediated cell capture. The etched silicon surface of the V-BioChip was coated with biotinylated polyethyleleglycol (biotin-PEG). After preincubating with biotinylated anti-EpCAM and anti-Ncadherin antibodies, CTCs were immobilized by streptavidin that strongly interacts with the biotin-PEG on the surface of the V-BioChip. Captured cells were visualized by secondary antibodies labeled with FITC (targeting anti-EpCAM) and Cy5 (targeting anti-CD13). (**b**) Scanning electron microscopy image of an immobilized cell on the V-BioChip. (**c**) Artificial intelligence assisted targeted cell recognition. Upper panel, identification of cell with FITC, TRITC, Cy5, and DAPI fluorescent signal from the V-BioChip. Lower panel, the fluorescent staining of an identified CTC.

CTCs were captured by the automated Cell Reveal^TM^ system, which is an automated silicon-based nano structured microfluidics we had previously reported [23,24,25]. Eight ml of peripheral blood were collected from the patients into BD vacutainer ACD Solution A blood collection tube (Becton Dickinson, Franklin Lakes, NJ, USA). PBMCs were isolated from the whole blood using Histopaque-1077 (Sigma-Aldrich, St. Louis, MO, USA). Isolated PBMCs were preincubated with biotinylated anti-EpCAM antibody and biotinylated anti-N-Cadherin antibody at 37 °C for 45 min [26] (Figure 1a). The mixture was then injected into the V-BioChip (CytoAurora Inc., HsinChu, Taiwan) which is a silicon-based chip etched with metal-assisted chemicals with matrix-arranged nano-pillar structure on the capture surface to increase the binding efficiency. The surface of the chip has been modified by silane deposition and coated with biotinylated polyethyleleglycol (Biotin-PEG) for better biocompatibility to reduce cell damage and as capture anchorage for streptavidin interaction. The strong interaction between biotin and streptavidin can immobilize the antibodies labeled CTCs on to the surface of the chip (Figure 1b). The captured cells were washed and stained with secondary antibodies labeled with fluorescent dyes (FITC, TRITC, and Cy5) and DAPI for nuclear visualization (Figure 1c).

**Figure 2:**
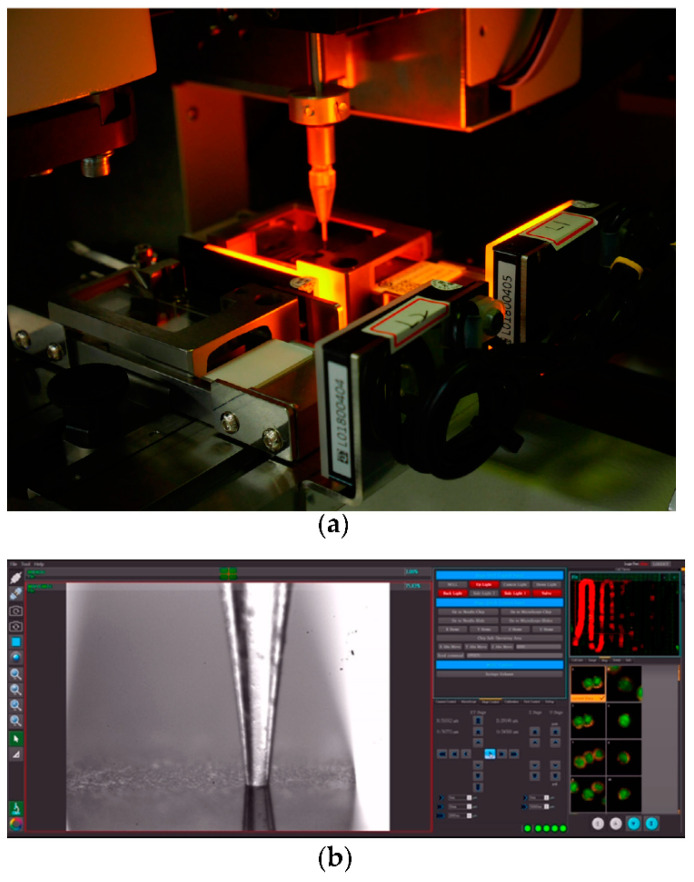
An automated cell retrieval device. (**a**) The cell retrieval platform and capture glass capillary tube. (**b**) Computer assisted cell capture operation. The capture glass capillary tube was monitored with high-resolution camera.

Captured CTCs were scanned by CytoAcqImages system (CytoAurora Inc., HsinChu, Taiwan). The scanning procedure was controlled by the cell analysis tools system for accurate positioning of correct fluorescent labeled cells. For each patient, all labeled cells were recovered by the cell picker system and pooled in 4 uL of TE buffer for further WGA (Figure 2).

### 2.4. Whole Genome Amplification (WGA)

WGA was performed for the captured CTCs using PicoPLEX Gold Single Cell DNA-seq kit (Takara Bio, Mountain View, CA, USA) according to the manufacturer’s manual. Amplified DNA fragments were purified with QIAquick PCR Purification kit (QIAGEN, Hilden, Germany). The fragment sizes were analyzed using Bioanalyzer 2100 (Agilent, Santa Clara, CA, USA), and the DNA concentrations were determined using Qubit dsDNA High Sensitivity Assay kit (Life technologies, Carlsbad, CA, USA).

### 2.5. Whole Exome Sequencing

Approximately 200 ng of genomic DNA from tumor tissues and matched germline controls (PBMCs) were subjected to ultrasonic fragmentation by Covaris S220 sonicator (Covaris, Woburn, MA, USA) to obtain a DNA fragment size ranging from 200 to 500 base pairs (bps). Fragmented genomic DNA was ligated to adapters containing sample specific indexing barcode (Illumina, San Diego, CA, USA) to construct a DNA library. Library construction was also performed for CTCs but the fragmentation was skipped because the amplicon size of WGA from CTCs was already in the desired range. After 8–12 cycles of polymerase chain reaction amplification, exonic region of input DNA samples were enriched by hybridization with Agilent SureSelect Human v6 probes (Agilent, Santa Clara, CA, USA). The enriched DNA were subjected to Illumina NovaSeq 4000 (Illumina, San Diego, CA, USA) for NGS with a 2 × 150 bp format. The average coverage depths of captured region were estimated 300× on tumor tissues, and 100× on CTCs and germline controls.

### 2.6. Exome Variation Analysis

The sequencing result was obtained in fastq farmat and aligned to human genome (GRCh38.p12) by bwa-mem aligner (version 0.7.17). The mapped bam files were analyzed by the Genome Analysis Toolkit (GATK, version 4.1.9.0) and followed the best practice proposed by the Broad institute. For germline variation detection, we focused on the important 544 cancer genes by utilizing the gene list of commercially available resources provided from Illumina TSO500 [27] and Roche Foundation Medicine Foundation One CDx [28]. The union gene list of both products was interrogated in our study, covering well-studied cancer driver genes and druggable targets. The tumor tissues/CTCs specific variants were called by mutect module provided by GATK (version 4.1.9.0). The result vcf files were annotated by several public resources including Ensembl (https://www.ensembl.org/index.html) (accessed on 15 May 2021), ClinVar (https://www.ncbi.nlm.nih.gov/clinvar/) (accessed on 15 May 2021), UCSC (https://genome.ucsc.edu/) (accessed on 15 May 2021), dbNSFP35a (https://sites.google.com/site/jpopgen/dbNSFP) (accessed on 15 May 2021), COSMIC (https://cancer.sanger.ac.uk/cosmic) (accessed on 15 May 2021), VEP (https://www.ensembl.org/info/docs/tools/vep/index.html) (accessed on 15 May 2021), 1000 genomes project (https://www.internationalgenome.org/) (accessed on 15 May 2021), ExAC (https://gnomad.broadinstitute.org/) (accessed on 15 May 2021), and gnomAD (https://gnomad.broadinstitute.org/) (accessed on 15 May 2021) to further classify the variation impact.

## 3. Results

### 3.1. CTCs Capture and Recovery

There are 6 and 8 cells being captured from 4 mL of peripheral bloods for Patient 1 and Patient 2, respectively, via the Cell Reveal^TM^ system. The chelating and identifying antibodies for EOC are EpCAM and CD13. Thus, all these cells are EpCAM(+)/CD13(+). Five out of the 6 CTCs captured from Patient 1 and all the 8 CTCs captured from Patient 2 were recovered for subsequent genetic analyses.

### 3.2. Capture Efficiency and Uniformity of WES

The sequencing coverages on the 6 samples (CTCs, tumor, and germline control/ PBMCs of Patient 1 and Patient 2) for WES are all greater than 100× (Supplementary Materials File S1) which is enough for germline variation discovery. In tumor samples, the coverages were around 300× that is optimal for the detection of minor somatic variations. For CTCs samples that comprise only 5~8 cells, sequencing depth greater than 100× is sufficient for variant detection, even only 1 cell with heterozygote mutation.

Analysis of the capture uniformity of samples, the genomic DNA with regular library construction protocol (tumor and PBMCs) showed around 3% of non-coverage rate, and DNA with WGA (CTCs) showed around 11% of non-coverage rate (Supplementary Materials File S1). Tumor and PBMCs had greater than 50% region with at least 100× coverage. On the contrary, the CTCs had lower coverage rate, approximately 40% with 50× and 30% with 100× (Supplementary Materials File S1).

### 3.3. Germline Variations in PBMCs

**Figure 3:**
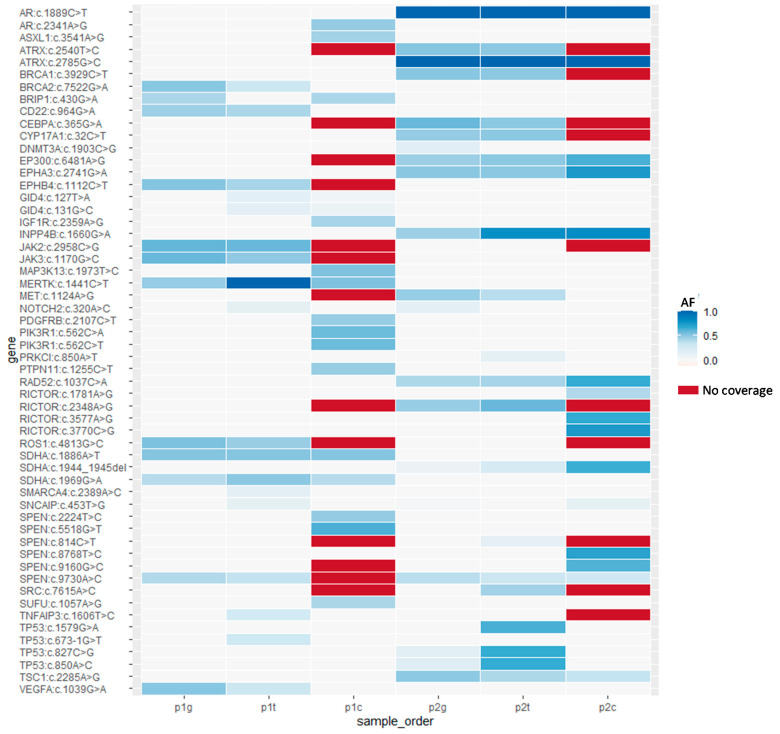
Overview of the genetic variations detected by whole exome sequencing in the two patients with epithelial ovarian cancer (EOC). The p1g, p1t, and p1c indicate germline controls/peripheral blood mononuclear cells, tumors, and CTCs respectively from Patient 1. The p2g, p2t, and p2c are the identical sample order of Patient 1 from Patient 2. The vertical axis indicates the identified variation site for each cancer driver gene. The color scale from white to dark blue represent the allele frequency (AF) of variations. Red color indicated the no sequencing read coverage.

A total of 56 genetic variations were detected in samples of PMBCs, primary tumors, and CTCs from the 2 patients with EOC (Supplementary Materials File S2). Of which, 28 germline variations in 25 genes were identified in PBMCs of the 2 patients (Figure 3 and Table 1). According to the WES results of tumors and CTCs, these variations were classified into 4 categories (Category I_g_, II_g_, III_g_, and IV_g_) (Table 1). In Category I_g_, variations were identically called in tumors and CTCs. Out of the 28 variations, 2 in *AR* and *ATRX* were called as homozygote, and 10 in *CD22*, *EP300*, *EPHA3*, *INPP4B*, *RAD52*, *SDHA*, *SPEN* and *TSC1* were called as heterozygote in PBMCs, tumors, and CTCs. In Category II_g_, variations were identically called in tumors but showed no call in CTCs. Nine variations in *ATRX*, *BRCA1*, *CEBPA*, *CYP17A1*, *EPHB4*, *JAK2*, *JAK3*, *RICTOR*, and *ROS1* were called as heterozygote in PBMCs and tumors, but the read coverage of these variations in CTCs were missing. In Category III_g_, variations were identically called in tumors but not detected in CTCs. Four variations in *BRCA2*, *MET*, *TP53*, and *VEGFA* were called as heterozygote in PBMCs and tumors but revealed wild-type in CTCs. In Category IV_g_, variations were not classified into the 3 categories mentioned above. Two variations in *BRIP1* and *DNMT3A* were called as heterozygote in PBMCs and tumors, but were called as heterozygote and wide-type respectively in CTCs. One variation in *MERTK* was called as heterozygote in PBMCs and CTCs, but was called as homozygote in tumors (Table 1). Overall, the germline variations detected in PBMCs revealed high heterogeneity in primary tumors and CTCs in terms of the variation distribution and frequency (Figure 3).

### 3.4. Somatic Mutations in Tumors

Twelve somatic mutations in 9 genes were identified in tumors but not in germline controls (Figure 3 and Table 2). According to the calling situation in CTCs, these somatic mutations were classified into 3 categories (Category I_t_, II_t_, and III_t_) (Table 2). In Category I_t_, somatic mutations can be also detected in CTCs. Out of the 12 somatic mutations, only 2 in *GID4* belong to this category. The remining 10 somatic mutations were not detected in CTCs. The Category II_t_ include 8 somatic mutations in *NOTCH2*, *PRKCI*, *SMARCA4*, *SNCAIP*, *TNFAIP3*, and *TP53* that were detected in tumors only, but remained wild type in CTCs. The Category III_t_ include 2 somatic mutations in *SPEN* and *SRC*. However, there is no read coverage in these mutations from the corresponding CTCs samples (Table 2). *NOTCH2*, *TP53*, and *SRC* are components of thyroid hormone signaling (Table 2). *NOTCH2* is categorized by the Gene Ontology biological process with a “negative regulation of transcription by RNA Polymerase II” (GO:0000122). *TP53* is a well-known tumor suppressor gene with cell cycle arresting and promoting apoptosis upon detection of genomic DNA damage. Two *TP53* mutations c.673-1G>T (rs878854073) and c.1579G>A (rs786202082) were classified as pathogenic in the ClinVar database. Another *TP53* mutation c.827C>G was considered as uncertain significance. Both *TP53* c.827C>G (p.A276G) and c.1579G>A (p.E527K) mutations locate within the DNA binding domain of TP53 protein and presumably have deleterious impacts on protein functions. The *SRC* c.7615A>C mutation (rs879255268) is recorded as pathogenic or likely-pathogenic in ClinVar. SRC and PRKCI proteins also participates in tight junction and platelet activation pathways, implying important roles on cell adhesion and proliferation.

### 3.5. Somatic Mutations in CTCs

Seventeen somatic mutations in 11 genes (*AR*, *ASXL1*, *IFG1R*, *MAP3K13*, *PDGFRB*, *PIK3R1*, *PTPN11*, *RICTOR*, *SNCAIP*, *SPEN*, and *SUFU*) were restrictively detected in captured CTCs, but not in the corresponding tumor samples (Figure 3 and Table 3). All 17 variations have not been reported in the ClinVar database but most genes play roles in the carcinogenesis. *AR*, *IGF1R*, *PDGFRB*, *PIK3R1*, and *SUFU* involved in KEGG pathways in cancer. *MAP3K13*, *PDGFRB*, *PIK3R1*, and *PTPN11* involved in Ras signaling pathway. Other cancer related pathways including proteoglycans in cancer, focal adhesion, Rap1 signaling pathway, PI3K/Akt pathway contain 2 or more genes with CTCs mutations, including *IGF1R*, *PDGFRB*, *PIK3R1*, and *PTPN11*. Since only 5 ~ 8 EpCAM(+)/CD13(+) positive CTCs were analyzed for each patient, clonal effect may affect the gross allele frequency. Supposedly only one out of 8 cells bear with a de novo heterozygote mutation, a minimal allele frequency of 0.06 (1/16) is expected. Therefore, a filter criterion for any called mutations with allele frequency >0.05 in CTCs sequencing results is chosen.

## 4. Discussion

In this study, CTCs from two patients with EOC were successfully isolated by an automated Cell Reveal^TM^ system [23,24,25]. Genomic profiles of CTCs as well as that of primary tumors and germline controls (PBMCs) were further examined and compared by WES. It is unexpected to find that CTCs harbor large number of private mutations that were not detected in tumor tissues, in contrast to the general assumption that CTCs are cells detached from primary tumor mass and should have similar or more mutation profiles than identified in tumor tissue. Furthermore, in patients’ tumors, several known pathogenic somatic mutations identified in *TP53* (c.673-1G>T (rs878854073) in Patient 1; SRC c.7615A>C (rs879255268), TP53 c.827C>G (rs786202082) in Patient 2) are supposedly cancer driver mutations, but they were not detected in CTCs. Aa a result, CTCs seem to be a specialized population raised intrinsically, as confirmed by the germline variation examination.

Whether CTCs will obtain key malignant mutations and rehabilitate in distant body part needs to be better understood. According to our experiences on preimplantation genetic testing [29,30,31,32,33], WGA process can successfully obtain enough genomic DNA for genetic analyses including array comparative genomic hybridization, quantitative polymerase chain reaction, or even whole genome sequencing or WES in single cell level. WES of single CTC was previously performed only in few cancer studies [34]. However, the quality of signal from single cell is still not as stable or clean as plural (two or more) cells. For better and accurate diagnosis, we pooled all the captured CTCs as starting materials for WGA and subsequently genetic analyses.

It is interesting to note highly variable allele frequencies between primary tumor and plural CTCs results. We, therefore, proposed a novel model to test if the phenomenon is caused by ADO in plural CTCs or tumor heterogeneity. If all possible genotypes were considered, we can have following number of combinations:

$$H_{n}^{3}=C_{n}^{n+3-1}=C_{3-1}^{n+3-1}=\frac{\left[n+\left(3-1\right)\right]!}{n!\times \left(3-1\right)!}=\frac{\left(n+2\right)!}{n!\times 2}$$

where H means repeat combination, 3 means 3 genotypes for diploid genes (homozygous wild type: AA, heterozygous mutation: Aa, and homozygous mutation: aa), and n means the total captured cells.

If such mutations occur only at a subset of CTCs or tumor, there will be $N_{1}$ cells to retain for AA, $N_{2}$ cells got the mutation and became Aa. It is unlikely that aa would occur since the probability of two independent episodes of mutation to co-occur is extremely low. If we ignore the loss of heterozygosity event, then the number of total captured CTCs (*N_t_*) can be expected as $N_{t}=N_{1}+N_{2}$.

The overall allele frequency for wild type (A) and mutant (a) allele may have  2n+1 possible combinations. The allele frequency of A (*F_A_*): $F_{A}=\frac{\left(N_{1}\times 2+N_{2}\times 1\right)}{2\times N_{t}}$ and then the allele frequency of a (*F_a_*): $F_{a}=\frac{N_{2}\;}{2\times N_{t}}$.

We further use the limited sets of combination of ($N_{1}$, $\;N_{2}$), of which $N_{1}+N_{2\;}=N_{t}$, to calculate if any combination fits our WES data. If the theoretical values perfectly match the experimental data, then it is possible that tumor heterogeneity exists, and there was no null amplification/ADO. However, if no combination can match the WES data, and we then try if ($N_{1}-n_{1}$ and $\;N_{2}-n_{2}$) can be matched (where $n_{1}$ and $n_{2}$ denote the number of AA and Aa cells to have null amplification/ADO during the experimental process), and null amplification/ADO will be inferred. Namely, if *n*_1_ or *n*_2_ ≥ 1 then it is likely that null amplification/ADO had occurred.

Considering the WES results in Patient 1, 5 CTCs were collected. If 1 out of 5 cells has heterozygote mutation at a specific locus, the theoretical allele frequency (AF) for mutant allele (*F_a_*) is 0.1. The theoretical *F_a_* frequencies may be $F_{a}=\frac{N_{2}\;}{2\times N_{t}}$, where $N_{2}=0\;to\;N_{t}.\;$

If all cells have heterozygote mutation at this locus, the ideal AF is 0.5. The overall AF can be detected by variant caller. Most of detected mutant AFs in both patients were close to 0.5 (0.4 ~ 0.6), indicating almost all cells were bearing heterozygote mutations. If genomic DNA from only 4 out of 5 cells were successfully amplified, the theoretical mutant AF may be 0.125 for 1 heterozygote cell, 0.25 for 2 heterozygote cells and so on. In our data from Patient 1, we detected the AF of *PIK3R1* c.562C>T is 0.08, indicating only 1 out of 5 CTCs had heterozygote mutation (theoretical is 0.1). In Patient 2, we also detected the AF of *SNCAIP* c.453T>G is 0.12, indicating 2 out of 8 CTCs had heterozygote mutations (theoretical AF is 0.125). This estimation also indicates the mutation event in Patient 1 came from 5 CTCs not 4 or fewer since the AF (0.08) is lower than the estimated level (0.1 for $N_{t}=5$, 0.125 for $N_{t}=4$, and 0.33 for $N_{t}=3$).

For AF greater than 0.7, the possible reason may result from ADO of wild type allele or homozygote mutation presented in some CTCs. In our result, we did not identify extremely high frequency locus (>0.85), therefore the slightly higher AF (0.7 ~ 0.85) may come from the combination of preferential amplification, ADO, and some homozygote mutations. If we control the input cell number for no more than 10 cells, ~5% AF is easy to be detected for only 1 cell has de novo heterozygote mutation. Most of the AF in CTCs are close to 0.5, indicating the ADO was not a major problem in our cases.

Null amplification did occur at some regions consistently, indicating the limitation of our current PicoPLEX-based WGA protocol. Since we are targeting more than 500 important tumorigenesis related genes, for a better reproducibility and comprehensiveness, it is better to use a pan-exome amplification protocol instead of using customized designs of oligos specific for limited targeting genes. Whether the utility of other WGA protocols or introducing molecular techniques like unique molecule index (UMI) can improve such amplification errors warrant further study [34,35].

## 5. Conclusions

Our results demonstrated that the Cell Reveal^TM^ platform can capture CTCs from stage I EOC patients and recovered the CTCs for subsequent genomic analyses. Our results also showed ADO is unlikely the major problem to cause the highly variable allele frequencies between primary tumor and plural CTCs results in our cases. This study can pave the way for future large-scale researches to further dissect the oncogenomics of OC and can contribute to the understanding of tumorigenesis and the corresponding treatments.

## Figures and Tables

**Table 1:** Germline variations detected in peripheral blood mononuclear cells (PBMCs) of the two patients with epithelial ovarian cancer (EOC).

Gene	Variation	Patient	PBMCs	Tumor	CTCs
Category I_g_					
*AR*	c.1889C>T	p2	Homo	Homo	Homo
*ATRX*	c.2785G>C	p2	Homo	Homo	Homo
*CD22*	c.964G>A	p1	Het	Het	Het
*EP300*	c.6481A>G	p2	Het	Het	Het
*EPHA3*	c.2741G>A	p2	Het	Het	Het
*INPP4B*	c.1660G>A	p2	Het	Het	Het
*RAD52*	c.1037C>A	p2	Het	Het	Het
*SDHA*	c.1886A>T	p1	Het	Het	Het
*SDHA*	c.1969G>A	p1	Het	Het	Het
*SDHA*	c.1944_1945del	p2	Het	Het	Het
*SPEN*	c.9730A>C	p1 and p2	Het	Het	Het
*TSC1*	c.2285A>G	p2	Het	Het	Het
Category II_g_					
*ATRX*	c.2540T>C	p2	Het	Het	NC
*BRCA1*	c.3929C>T	p2	Het	Het	NC
*CEBPA*	c.365G>A	p2	Het	Het	NC
*CYP17A1*	c.32C>T	p2	Het	Het	NC
*EPHB4*	c.1112C>T	p1	Het	Het	NC
*JAK2*	c.2958C>G	p1	Het	Het	NC
*JAK3*	c.1170G>C	p1	Het	Het	NC
*RICTOR*	c.2348A>G	p2	Het	Het	NC
*ROS1*	c.4813G>C	p1	Het	Het	NC
Category III_g_					
*BRCA2*	c.7522G>A	p1	Het	Het	Wt
*MET*	c.1124A>G	p2	Het	Het	Wt
*TP53*	c.850A>C	p2	Het	Het	Wt
*VEGFA*	c.1039G>A	p1	Het	Het	Wt
Category IV_g_					
*BRIP1*	c.430G>A	p1	Het	Wt	Het
*DNMT3A*	c.1903C>G	p2	Het	Wt	Wt
*MERTK*	c.1441C>T	p1	Het	Homo	Het

p1, Patient 1; p2, Patient 2; Homo, homozygote; Het, heterozygote; Wt, wild type; NC, no call.

**Table 2:** Somatic mutations detected in tumors of the two EOC patients.

Gene	Variation	Patient	Tumor AF	CTCs AF	Pathway
Category I_t_					
*GID4*	c.127T>A	p1	0.16	0.06	NA
*GID4*	c.131G>C	p1	0.15	0.086	
Category II_t_					
*NOTCH2*	c.320A>C	p1	0.13	Wt	Thyroid hormone signaling
*PRKCI*	c.850A>T	p2	0.13	Wt	Tight junction
*SMARCA4*	c.2389A>C	p1	0.15	Wt	NA
*SNCAIP*	c.453T>G	p1	0.12	Wt	NA
*TNFAIP3*	c.1606T>C	p1	0.26	Wt	NA
*TP53*	c.673-1G>T	p1	0.3	Wt	Thyroid hormone signaling
*TP53*	c.827C>G	p2	0.66	Wt	
*TP53*	c.1579G>A	p2	0.64	Wt	
Category III_t_					
*SPEN*	c.814C>T	p2	0.14	NA	NA
*SRC*	c.7615A>C	p2	0.45	NA	Tight junction Thyroid hormone signaling Platelet activation

AF, allele frequency; p1, Patient 1; p2, Patient 2; Wt, wild type; NA, not available.

**Table 3:** Somatic mutations detected in circulating tumor cells (CTCs) of the two EOC patients.

Gene	Variation	Patient	Tumor	CTCs AF	Pathway
*AR*	c.2341A>G	p1	Wt	0.48	Pathways in cancer
*ASXL1*	c.3541A>G	p1	Wt	0.44	NA
*IGF1R*	c.2359A>G	p1	Wt	0.43	Pathways in cancer Ras signaling pathway Proteoglycans in cancer Focal adhesion Rap1 signaling pathway PI3K/Akt pathway Progesterone-mediated Oocyte maturation
*MAP3K13*	c.1973T>C	p1	Wt	0.53	NA
*PDGFRB*	c.2107C>T	p1	Wt	0.47	Pathways in cancer Ras signaling pathway Focal adhesion Rap1 signaling pathway PI3K/Akt pathway mTOR pathway
*PIK3R1*	c.562C>A	p1	Wt	0.45	Pathways in cancer Ras signaling pathway Proteoglycans in cancer Focal adhesion Rap1 signaling pathway PI3K/Akt pathway Progesterone-mediated Oocyte maturation
	c.562C>T	p1	Wt	0.08	
*PTPN11*	c.1255C>T	p1	Wt	0.47	Ras signaling pathway Proteoglycans in cancer
*RICTOR*	c.3770C>G	p1	Wt	0.75	mTOR pathway
	c.3577A>G	p2	Wt	0.67	
	c.1781A>G	p2	Wt	0.41	
*SNCAIP*	c.453T>G	p2	Wt	0.12	NA
*SPEN*	c.5518G>T	p1	Wt	0.63	NA
	c.2224T>C	p1	Wt	0.48	
	c.8768T>C	p2	Wt	0.71	
	c.9160G>C	p2	Wt	0.62	
*SUFU*	c.1057A>G	p1	Wt	0.42	Pathways in cancer

AF, allele frequency; p1, Patient 1; p2, Patient 2; Wt, wild type; NA, not available.

## Data Availability

Data are available upon request.

## Supplementary Materials

The following are available online at https://www.mdpi.com/article/10.3390/diagnostics11061102/s1, File S1: whole exome sequencing (WES) on primary tumor, CTCs, and matched germline control (peripheral blood mononuclear cells, PBMCs). File S2: comparisons of the genetic variations detected in primary tumor, CTCs, and PBMCs of the 2 patients with EOC.
