# Supplementary material for: Comparison of Genetic Profiling between Primary Tumor and Circulating Tumor Cells Captured by Microfluidics in Epithelial Ovarian Cancer: Tumor Heterogeneity or Allele Dropout?
Source: Diagnostics (Basel). 2021 Jun 16;11(6):1102. doi: 10.3390/diagnostics11061102 (PMC8234832; doi:10.3390/diagnostics11061102)
Supplement: Supplementary file 1 [file diagnostics-11-01102-s001.zip › File S1_Whole exome sequencing (WES) on primary tumor, CTCs, and matched germline control (peripheral blood mononuclear cells, PBMCs).pdf]

File S1

Whole exome sequencing (WES) on primary tumor, CTCs, and matched germline control (peripheral blood mononuclear cells, PBMCs)

To investigate the capture efficiency and uniformity in exonic region, we first investigate the mean target coverage on all 6 samples (Figure A1a). We found the mean coverage was greater than 100× in all samples, indicating the total sequencing depth is enough for germline variation discovery. In tumor part, considering the heterogeneity of tumor tissue, around 300× coverage is optimal for the detection sensitivity of minor somatic variations. Since CTCs samples only comprised of 5~8 cells, sequencing depth greater than 100× is sufficient for variant detection for even 1 cell with heterozygote mutation. As for the uniformity of all samples, we found genomic DNA with regular library construction protocol (tumor and blood) showed around 3% of non-coverage rate; while DNA with whole genome amplification showed around 11% of non-coverage rate consistently (Figure A1b). We than investigated the coverage statistics from percentage of targeting region with 1x coverage to 100x (Figure A1c). We found both tumor and blood samples had greater than 50% region with at least 100× coverage; however, the CTCs samples had lower coverage rate (around 40% with 50×, 30% with 100×). We than summarized total coverage reads on 3 important cancer genes for each sample. We found on *ERBB2* and *MET*, all samples had good coverage depth; however on *PTEN*, both CTCs coverage rate is lower than tumor/blood samples, indicating some uneven amplification occurred in the whole genome amplification steps (Figure A1d). To study whether DNA copy number was altered after whole genome amplification, we analyzed the gross DNA copy number variation by comparing tumor and CTCs with match blood samples. In Figure A1e, we found slight but not prominent copy number alteration (within ±2 folds) on patient 1; whereas In Figure A1g, regions on chr5, chr11 had significant amplification were detected. In CTCs samples from both patients, although some targeted region had no signal, generally the copy number was evenly distributed across the genome (Figure A1f, A1h). We then visualized the alignment on some important cancer genes using Integrated Genome Viewer (IGV) (Figure A2). On *TP53*, we found 3 targeting regions for both CTCs (Figure A2a, blue circles) on both CTCs samples had fewer coverage rate than tumor/blood counterpart. However, the read number still sufficient for variant calling. On *ERBB2*, we found 2 regions with fewer coverage rate consistently (Figure A2b). On *PTEN* and *MET*, we found 1 region with zero amplification and 1 region with fewer coverage rate (Figure A2c and A2d).

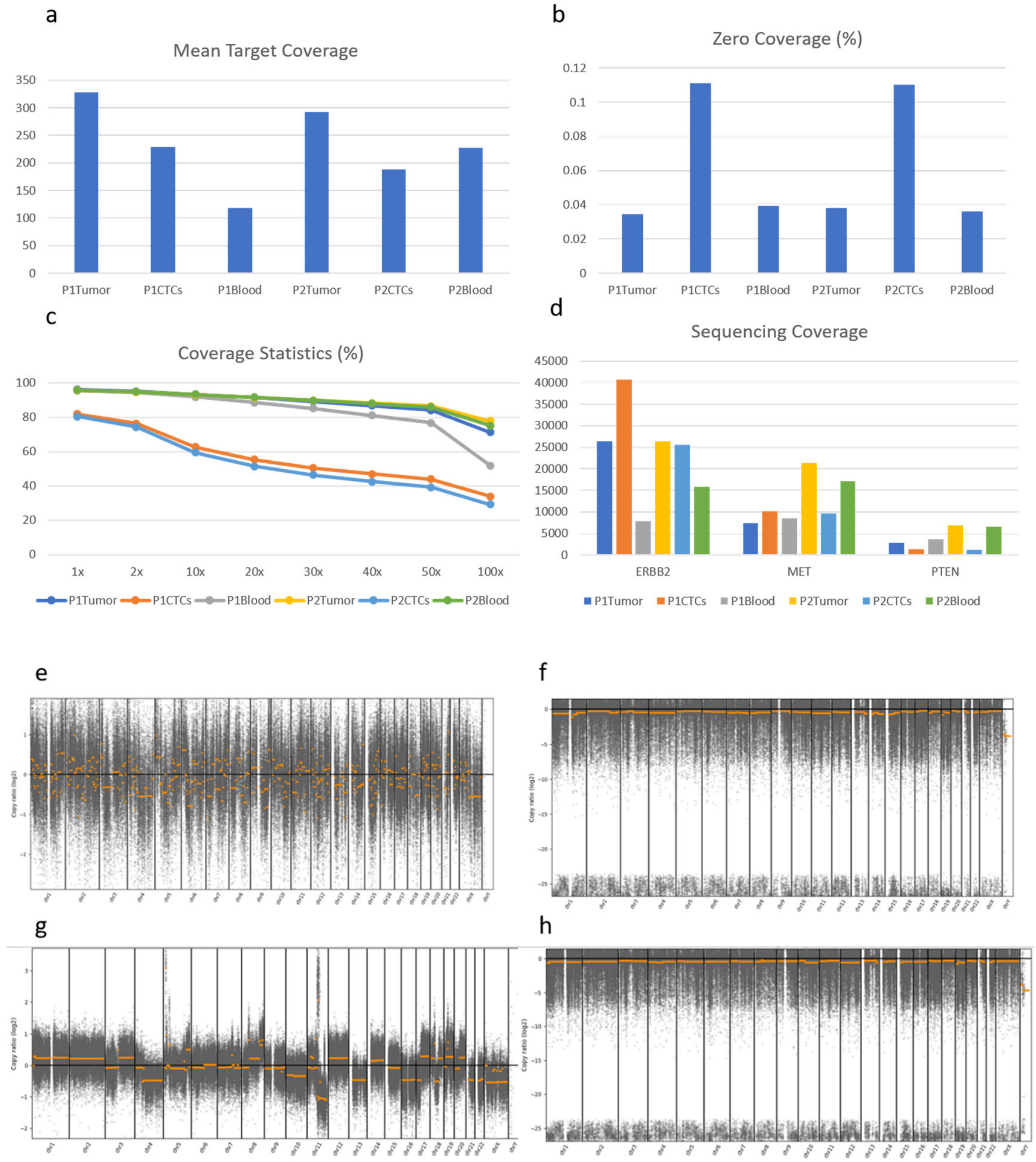

Figure A1 Overview of the whole exome sequencing (WES) for tumor, CTCs and germline control/white blood cells from 2 patients with epithelial ovarian cancer (EOC). (a) The mean coverage of 6 samples (CTCs, tumor, and germline control/white blood cells of Patient 1 and Patient 2). Y-axis indicates the sequencing coverage of the designed targeting region. (b) Zero coverage percentage of 6 samples. (c) The gross statistics in percentage from 1x to 100x coverage. (d) The sequencing coverage on 3 important cancer genes of 6 samples. (e-h) Copy number analysis for (e) patient 1 tumor v.s. matched blood, (f) patient 2 CTCs v.s. matched blood, (g) patient 2 tumor v.s. matched blood, (h) patient 2 CTCs v.s. matched blood.

a

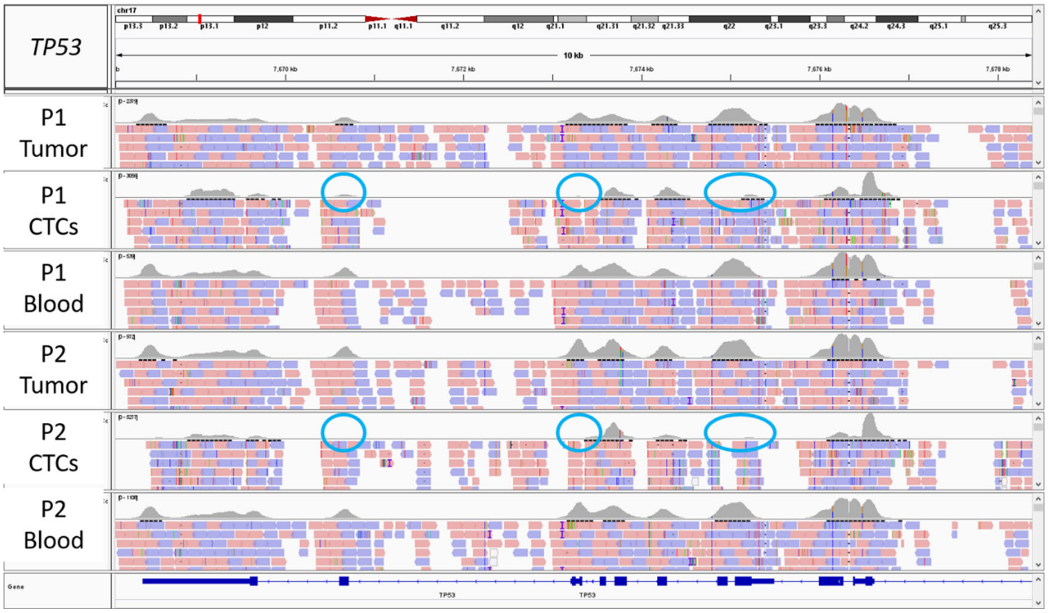
TP53
chr17
10 kb
P1
Tumor
P1
CTCs
P1
Blood
P2
Tumor
P2
CTCs
P2
Blood
Gene

b

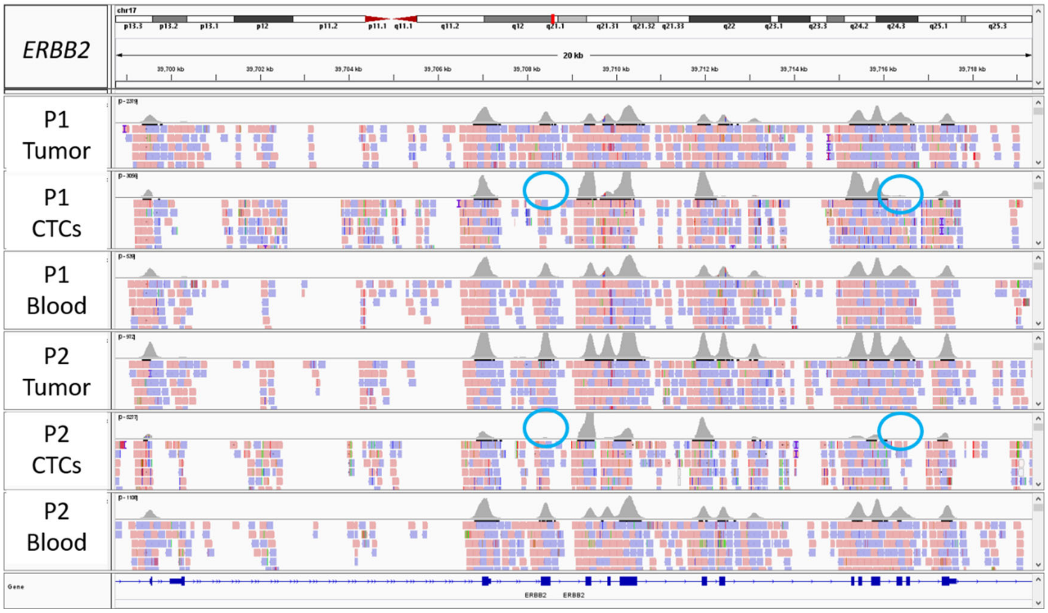
ERBB2
chr17
20 kb
P1
Tumor
P1
CTCs
P1
Blood
P2
Tumor
P2
CTCs
P2
Blood
Gene

c

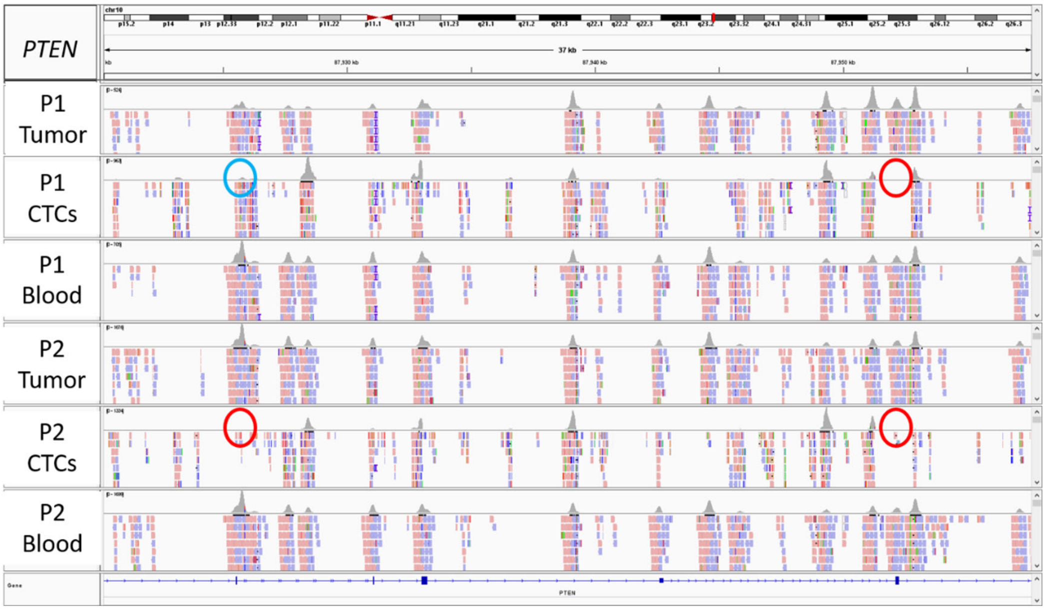

d

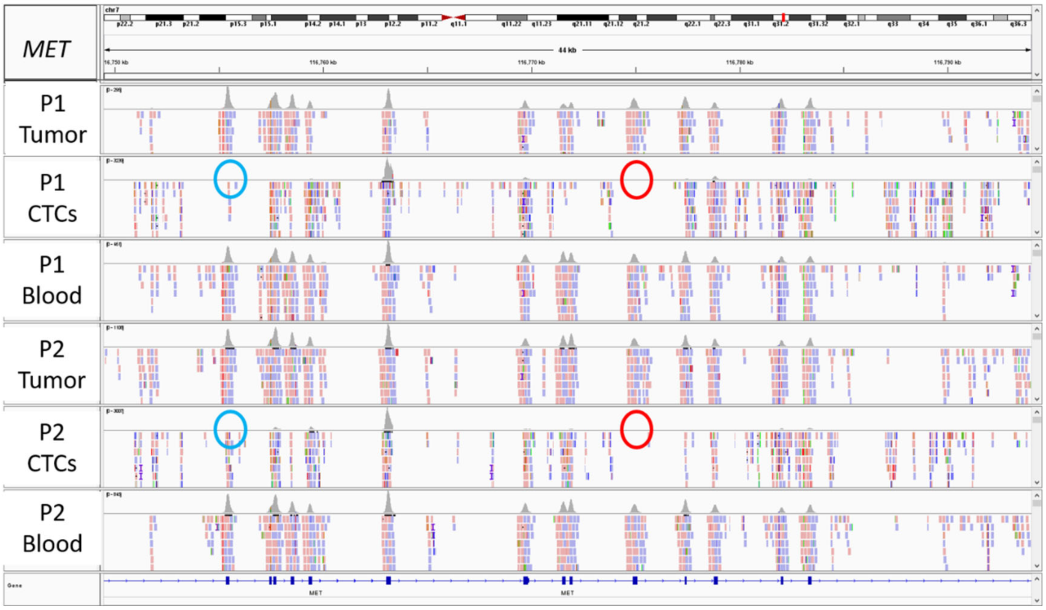

Figure A2 Exemplified gene alignment by the Integrative Genomics Viewer. Sequencing reads mapped to genes of (a) *TP53* (b) *ERBB2* (c) *PTEN* and (d) *MET*. Blue circles indicating regions with fewer read coverage in CTCs than in blood and tumor. Red circles indicating regions with almost no read coverage in CTCs.
