# Supplementary material for: Comparison of Genetic Profiling between Primary Tumor and Circulating Tumor Cells Captured by Microfluidics in Epithelial Ovarian Cancer: Tumor Heterogeneity or Allele Dropout?
Source: Diagnostics (Basel). 2021 Jun 16;11(6):1102. doi: 10.3390/diagnostics11061102 (PMC8234832; doi:10.3390/diagnostics11061102)
Supplement: Supplementary file 1 [file diagnostics-11-01102-s001.zip › File S2 Comparisons of the genetic variations detected in primary tumor, CTCs, and PBMCs of the 2 patients with EOC.pdf]

File S2

Comparisons of the genetic variations detected in primary tumor, CTCs, and PBMCs of the 2 patients with EOC

Table A1. Germline variations and somatic mutations detected in Patient 1 only (green), Patient 2 only (red), and both patients (yellow).

	**PBMCs**	**Tumor**	**CTCs**
**Germline variations**			
MERTK:c.1441C>T	0.48	1	0.53
SDHA:c.1886A>T	0.52	0.52	0.52
SDHA:c.1969G>A	0.4	0.5	0.4
VEGFA:c.1039G>A	0.51	0.32	0
ROS1:c.4813G>C	0.54	0.47	NA
EPHB4:c.1112C>T	0.52	0.43	NA
JAK2:c.2958C>G	0.6	0.58	NA
BRCA2:c.7522G>A	0.51	0.32	0
BRIP1:c.430G>A	0.42	0	0.42
JAK3:c.1170G>C	0.58	0.49	NA
CD22:c.964G>A	0.46	0.42	0
AR:c.1889C>T	1	1	1
ATRX:c.2785G>C	1	1	1
ATRX:c.2540T>C	0.52	0.5	NA
BRCA1:c.3929C>T	0.51	0.5	NA
CEBPA:c.365G>A	0.59	0.5	NA
CYP17A1:c.32C>T	0.48	0.5	NA
DNMT3A:c.1903C>G	0.18	0	0
EP300:c.6481A>G	0.46	0.5	0.64
EPHA3:c.2741G>A	0.51	0.5	0.75
INPP4B:c.1660G>A	0.46	0.79	0.81
MET:c.1124A>G	0.47	0.38	0
RAD52:c.1037C>A	0.42	0.43	0.66
RICTOR:c.2348A>G	0.47	0.58	NA
SDHA:c.1944_1945del	0.11	0.25	0.65
SPEN:c.9730A>C	0.4/0.38	0.35/0.32	NA/0.29
TP53:c.850A>C	0.19	0.66	0
TSC1:c.2285A>G	0.5	0.41	0.35

**Somatic mutations**			
NOTCH2:c.320A>C	0.15	0.13	0
SNCAIP:c.453T>G	0.04	0.12	0.12
TNFAIP3:c.1606T>C	0	0.26	0/NA
TP53:c.673-1G>T	0	0.3	0
GID4:c.127T>A	0	0.16	0.06
GID4:c.131G>C	0	0.15	0.086
SMARCA4:c.2389A>C	0	0.15	0
SPEN:c.814C>T	0	0.14	NA
PRKCI:c.850A>T	0	0.13	0
TP53:c.827C>G	0.17	0.66	0
TP53:c.1579G>A	0	0.64	0
SRC:c.7615A>C	0	0.45	NA
SPEN:c.5518G>T	0	0	0.63
PIK3R1:c.562C>A	0	0	0.57
SPEN:c.2224T>C	0	0	0.48
SUFU:c.1057A>G	0	0	0.42
AR:c.2341A>G	0	0	0.48
ASXL1:c.3541A>G	0	0	0.44
IGF1R:c.2359A>G	0	0	0.43
MAP3K13:c.1973T>C	0	0	0.53
PDGFRB:c.2107C>T	0	0	0.47
PTPN11:c.1255C>T	0	0	0.47
PIK3R1:c.562C>T	0	0	0.57
RICTOR:c.3770C>G	0	0	0.75
RICTOR:c.3577A>G	0	0	0.67
RICTOR:c.1781A>G	0	0	0.41
SPEN:c.8768T>C	0	0	0.71
SPEN:c.9160G>C	0	0	NA/0.62

Numbers indicated variation allele frequencies. 0: wide type; NA: no sequencing coverage on the given locus.
